# Assessment of weight bias among students and health professionals in medical radiation science. A protocol for a systematic review and meta-analysis

**DOI:** 10.1371/journal.pone.0345171

**Published:** 2026-03-25

**Authors:** Theresa O’ Donovan, Megan Brydon, Aisling Barry, Mark McEntee

**Affiliations:** 1 Medical Imaging and Radiation Therapy, School of Medicine, University College Cork, Cork, Ireland; 2 Department of Health and Wellness, Nova Scotia, Canada; 3 CancerResearch@UCC, School of Medicine, 4th Floor Western Gateway Building, Western Road, University College Cork, Cork, Ireland; 4 Department of Radiation Oncology, Cork University Hospital, Cork, Ireland; Independent Medical Researcher and Writer, UNITED KINGDOM OF GREAT BRITAIN AND NORTHERN IRELAND

## Abstract

Weight bias is pervasive in health care, contributing to adverse physical and psychological outcomes for affected individuals. While weight bias has been studied in various healthcare contexts, its presence and impact within medical radiation science remain underexplored. This systematic review aims to synthesise the existing literature on the assessment of weight bias in medical radiation science. This protocol is registered with PROSPERO (CRD42024506662). Comprehensive searches will be conducted across multiple electronic databases using predefined search strategies. Retrieved records will be imported into Covidence for title and abstract screening, followed by full-text review. Two independent reviewers will assess study eligibility and extract data. Methodological quality will be evaluated using a multi-tool appraisal approach tailored to the design of each study. Risk-of-bias will be assessed, and meta-biases will be explored through the inclusion of grey literature. Confidence in the cumulative evidence will be evaluated using the Grading of Recommendations Assessment, Development and Evaluation (GRADE) system. This systematic review protocol will provide a comprehensive synthesis of the quantitative assessment of weight bias among professionals and students in medical radiation science. By mapping how weight bias is measured, the findings may support awareness-raising efforts and inform development of strategies to reduce weight bias in education and clinical practice. Once complete, the review will be shared internationally via presentations or posters at conferences. It will be published in a peer-reviewed journal.

## Background and rationale

Globally, the average weight of the population is increasing. In 2022, the World Health Organisation (WHO) reported that 2.5 billion adults were “overweight”, and this figure is set to rise [1]. These projections will change the demographic profile of patients accessing healthcare services.

Weight bias refers to negative stereotypes, prejudicial attitudes, perceptions and judgements directed at individuals based on their body weight [2–4]. This type of bias can be implicit, which is described as unconscious bias or explicit where individuals consciously endorse such biases [5–8]. Implicit bias is most commonly measured in healthcare research using The Implicit Association Test [9]. This is a reaction-time-based measure where individuals are required to pair certain attributes with one group of people compared to another. Explicit bias is commonly assessed using a variety of self-report measures. A recent systematic review revealed 26 different measures in use to assess explicit bias in healthcare professionals [10].

Weight bias is common in all areas of society including the workplace, education, media and interpersonal relationships [11–15]. Individuals experience weight bias from a young age with teachers assigning lower grades to those with higher weight [12]. It can also manifest in the workplace, where individuals have been instructed to lose weight to keep their job or where job applications were rejected on the basis of weight [16,17].

Weight bias in healthcare is also pervasive. Bias towards individuals of higher weight is well established in multiple health care professions, even those who are involved in weight loss and weight management [18–34]. Research demonstrates that healthcare professionals often perceive people with higher weight as awkward, unattractive, and non-compliant [35]. The presence of weight bias and stigma within healthcare can impact patients negatively, causing a loss of trust in healthcare providers, as well as healthcare avoidance behaviours, such as reduced engagement with screening programs and delays in seeking treatment [36–41]. It may also contribute to lowered self-esteem, heightened anxiety, depression and chronic stress [42,43].

Explicit bias can manifest as misdiagnosis, altered clinical management, inadequate care, a lack of appropriate equipment (gowns, chairs, etc.), and inappropriate comments about weight [44–46]. Of greater concern is the insidious manifestation, such as health care professionals avoiding eye contact, not listening, engaging in shorter consultations or speaking in derogatory terms with other colleagues [39,47–53].

Evidence of weight bias has been documented within medical radiation science literature where articles with titles such as “what do we know about a big issue” have been published [54]. Individuals with higher body weight have been characterised as presenting challenges, causing inconvenience, or eliciting discomfort among practitioners [54–61].

Individuals who encounter professionals within medical radiation science are often in a vulnerable position, potentially undergoing investigations based on symptoms, or they are receiving treatment for a cancer diagnosis. There is a paucity of synthesised evidence focusing specifically on the assessment of weight bias in medical radiation science. The impact of weight bias within this sphere can lead to patient discomfort, reduced trust, and avoidance of care [49].

A review is warranted to systematically synthesise the research on the quantitative measurement of weight bias among professionals and trainees in medical radiation science. This systematic review aims to synthesise and critically appraise the evidence on quantitative tools (Intervention) used to assess weight bias (Outcome) among students and health professionals in medical radiation science (Population) within education, training and clinical practice contexts.

The aim of the protocol is to outline the methods that will be utilised in this systematic review.

## Methods

We will follow the Preferred Reporting Items for Systematic reviews and Meta-Analyses (PRISMA) guidelines [62].

### Information sources

A systematic search will be performed in five electronic databases: CINAHL Plus with Full Text (EBSCO), Embase, PubMed, PsycINFO (EBSCOHost) and Web of Science. Authors of conference abstracts will be contacted for access to full-text articles if available. Reference lists of articles retrieved during preliminary searches, individual authors, and key journals will be hand searched through Google Scholar.

### Search strategy

A systematic search of the literature on assessment of students and health professionals within medical radiation science will be conducted using five electronic databases (CINAHL, Embase, PubMed, PsycINFO [EBSCOHost] and Web of Science). Controlled vocabulary and keywords will be used to search for terms for medical radiation science health professionals and their students/trainees, obesity, bias and assessment. Keywords for each concept will be identified through a preliminary review of the literature. Guidance and advice will be sought from institutional librarian services to further refine the strategies. Searches will be combined using Boolean operators. No restrictions on the year of publication will be imposed. No language restrictions will be imposed. To overcome publication bias, citation chasing and grey literature will be searched. Searches will be updated before submission of the systematic review for publication. A search strategy has been included in a supplementary file.

### Study selection process

Studies will be selected based on eligibility criteria (Table 1). All eligible studies will be imported into Covidence systematic review software (Veritas Health Innovation, Melbourne, Australia) [63]. Two reviewers will independently assess each title and abstract against the inclusion and exclusion criteria. Once complete, we will conduct a full text review of the remaining articles. If eligibility remains unclear, we will contact study corresponding authors via email (max of two email attempts) to obtain additional information. Any disagreements will be discussed and resolved by consensus. A third party will be consulted if the first two reviewers cannot reach an agreement. We will document specific reasons for exclusion of studies.

**Table 1:** Eligibility Criteria.

	Inclusion Criteria	Exclusion Criteria
Study Design	Cross-sectional, Longitudinal, Prospective cohort,	Commentaries, Letters to editor, Case reports Reviews
Types of participants:	Human participants 18 years or older Students/trainees or professionals working within medical radiation science (including but not exclusively- Radiation Therapist (RTT), Radiation Therapist Technologist, Therapeutic Radiographer, Therapeutic Radiographer Practitioner, Medical Radiation Technologist, Medical Radiation Sciences Professional, Radiologic Technologist, Radiation Oncologist, Clinical Oncologist, Radiologist, Diagnostic Radiographer, Nuclear Medicine Technologist, MRI Technologist, Sonographer, Ultrasonographer, Ultrasound technologist).	Participants under 18 years of age Students registered to healthcare courses other than medical radiation science. Healthcare professionals practicing in areas outside of medical radiation science
Types of outcomes: Critical	Measures that directly reflect bias levels, such as scores from bias assessment tools e.g., Anti-Fat Attitudes Questionnaire [7], Fat Phobia Scale- short form (FPS) [6], The Beliefs About Obese Persons (BAOP) Scale Attitudes Toward Obese Persons (ATOP) Scale [5], Implicit Association Test (IAT) [64 65], or another appropriate quantitative tool.	Studies that do not use quantitative tools to report weight bias outcomes—either implicit or explicit

### Data extraction

Data extraction will be conducted by two reviewers independently. A data extraction tool will be developed for this review in Microsoft Excel (version 2506, Microsoft Corporation, Redmond, WA, USA). Included elements are informed by preliminary research in this area and the knowledge of research team. Covidence systematic review software (Veritas Health Innovation, Melbourne, Australia) will be used to manage and extract these data [63]. Data items to be collected are displayed in Table 2. Disagreements will be resolved through discussion and consultation with a third party where required (another researcher within the team).

**Table 2:** Data Items to be extracted.

Data Extraction
Study Characteristics:
• Journal
• Author(s)
• Year of publication
• Country/ geographical area study conducted.
• Study Title
• Study design (Qualitative v Quantitative v Mixed Methods)
• Duration (Start and end dates)
• Single v multi-centre
• Ethical approval (Yes/No)
Population Details:
• Inclusion Criteria
• Recruitment strategies
• Sampling
• Sample Size
• Age
• Gender
• BMI
• Professional/Student
• Years of clinical experience/ year of study
• Other relevant demographic details
Exposure:
• Data collection method(s) • Location(s) of data collection
• Method of analysis used
Outcome measures:
• Implicit bias- reaction time-based measures, mean differences, standardised mean difference (SMD) or effect sizes where applicable.
• Explicit bias: means and standard deviations.
• Effect size: mean differences or standardised mean differences (SMDs), SE, 95% Confidence Intervals (95% CI),
Results/Main findings related to instruments/tools used
Funding Sources
Conflicts of interest
Limitations

### Quality assessment

To assess the methodological quality of included studies, we will employ a multi-tool appraisal approach tailored to the specific design of each study. This strategy will ensure that each study type will be evaluated using criteria most appropriate to its methodological framework.

- Cross-sectional studies will be assessed using the CASP Cross-Sectional Checklist, focusing on sampling strategy, measurement validity, and risk of bias [66].
- Mixed-methods studies, where applicable, will be reviewed using the Mixed Methods Appraisal Tool (MMAT) [67] provided the study design aligns with MMAT’s criteria.

Two reviewers will independently appraise each study. Discrepancies will be resolved through discussion or consultation with a third reviewer. Appraisal results will be used to inform the synthesis and interpretation of findings, but no studies will be excluded solely based on quality scores.

### Data synthesis

A narrative synthesis will be undertaken, supported by a summary of findings and a data extraction table. Study characteristics will be analysed to facilitate comparisons.

For the studies where a meta-analysis is possible, pooled estimates will be calculated using a random-effects model. Effect sizes will be reported as standardised mean differences (Hedges g). Heterogeneity will be assessed with Cochrane’s Q and I^2^. If the Cochrane’s Q statistic suggests the presence of heterogeneity (*p* < 0.10), the degree of inconsistency across studies will be quantified using the I² statistic. In accordance with conventional interpretation guidelines, I² values will be categorised as low (0–40%), moderate (41–60%), and substantial heterogeneity [62] (61–100%).

### Subgroup and sensitivity analyses

Where sufficient data are available, we will conduct subgroup analyses to explore potential sources of heterogeneity in study findings. Subgroup analyses will be based on participant characteristics (e.g., gender, ethnicity, age, body mass index, profession, year of study or years of professional experience). Any meta-analysis will be completed using Comprehensive Meta-Analysis (CMA) version 4.0. Sensitivity analysis will test robustness by comparing fixed‑ vs. random‑effects models and restricting to validated measurement tools or rigorous qualitative methodologies.

### Assessment of Meta-bias(es)

To assess and reduce publication bias, a search of the grey literature will be conducted. If a meta-analysis is possible, then a funnel plot can be used to visually detect asymmetry, which is suggestive of publication bias. Any available protocols will be compared to final publications to overcome selective outcome reporting.

### Confidence in cumulative evidence

We will apply the Grading of recommendations Assessment, Development and Evaluation (GRADE) system to evaluate the certainty of evidence across outcomes [68]. A Summary of Findings (SOF) table will be developed for key outcomes using GRADE evidence profiles. A timeline of the project is provided (Table 3)

**Table 3:** Timeline of project.

Phase	Task	Deadline
1	Planning and Protocol Development	Completed
2	Literature Search	December 15^th^- 20^th^
3	Screening and Selection	December 22^nd^-February 2^nd^
4	Data Extraction	February 3^rd^ – 15^th^
5	Data Analysis and Synthesis	February 16^th^-28^th^
6	Writing and Reporting	March 1^st^-15^th^
7	Dissemination and Follow-up	March 16^th^-31^s^^t^

### Patient and public involvement

This review will not involve patients or members of the public in its design, conduct, reporting, or dissemination. The study is based exclusively on previously published literature and does not include primary data collection or stakeholder engagement.

### Ethical considerations

As this study is a systematic review of published literature, it does not involve human participants, personal data, or interventions. Therefore, ethical approval was not required. All included studies will be assessed for ethical compliance as part of the appraisal process.

### Protocol deviation

Any deviations from the protocol that occur during the conduct of the review will be documented and reported in the final manuscript. The date of changes and rationale will be provided, and their impact on the process will be discussed transparently.

### Data sharing

Two reviewers will independently extract data (study characteristics, populations, outcomes, and risk-of-bias assessments) from eligible articles and grey literature. Covidence will be used to store and organise all data during the review. Data will be securely stored on institutional servers with regular backups. A pilot test of the extraction form will be conducted on a sample of studies to ensure clarity and consistency. Regular checks will be performed to verify accuracy and completeness of extracted data. Upon completion of the review, anonymised datasets and extraction forms will be made available via an institutional repository, subject to ethical and legal considerations. Metadata and documentation will be provided to facilitate reuse. Any changes will be documented and updated in PROSPERO with justification.

## Discussion

This will be the first systematic review to synthesise quantitative assessment of weight bias in medical radiation science, addressing a critical gap in the existing literature. Many studies involving medical radiation science students and health professionals have only emerged in the past three years and therefore have not been represented in previous reviews of healthcare professionals more broadly.

By examining how weight bias has been measured in medical radiation science contexts – where patient encounters frequently involve physical exposure and heightened vulnerability- this review has the potential to highlight an underexplored and ethically significant issue. Variability in study designs, populations and measurement tools may limit direct comparisons, nonetheless, efforts will be made to ensure rigour throughout the screening and extraction process.

## Conclusion

This systematic review will synthesise the quantitative assessment of weight bias among medical radiation science students and health professionals. By mapping how weight bias has been measured in this population and identifying existing gaps, the findings may support the development of future awareness initiatives and inform strategies aimed at reducing weight bias in both education and clinical settings. Ultimately this work has the potential to contribute to more equitable, patient-centred care in medical radiation science. On completion the findings will be disseminated through conference presentations, posters and publication in a peer-reviewed journal.

## Supporting information

S1 File S1_PRISMA-P.checklist. (DOCX)

S2 File S2 Search Strategy. (DOCX)
