# Supplementary material for: Assessment of weight bias among students and health professionals in medical radiation science. A protocol for a systematic review and meta-analysis
Source: PLoS One. 2026 Mar 25;21(3):e0345171. doi: 10.1371/journal.pone.0345171 (PMC13016337; doi:10.1371/journal.pone.0345171)
Supplement: S2 File — (DOCX) [file pone.0345171.s002.docx]

# Appendix: Search Strategy (PICO Framework)

| PICO Element | Search Concept | CINAHL Database |
| --- | --- | --- |
| Population (P) | Medical Radiation Science students and professionals | Controlled Vocabulary: MH (Radiologic Technologists+), MH (Radiation Therapy Technologists), MH (Ultrasound Technologists), MH (Radiologists), MH (Radiation Oncologists), MH (Students, Radiologic Technology)  Keywords:  XB (Radiographer* OR “radiologist tech*” OR “radiation therapist*” OR "radiation therapy tech*" OR radiologist* OR “radiation oncologist*” OR "clinical oncologist*" OR “medical radiation tech*” OR “MRT” OR “MRSP” OR “medical radiation sciences professional*” OR “medical radiation tech*” OR "x-ray tech*" OR “radiologic tech*” OR sonographer* OR ultrasonographer* OR "Ultrasound Technologist*" OR “nuclear medicine tech*” OR "magnetic resonance tech*" OR "MRI tech*" OR mammographer* OR "student radiographer*" OR "student radiation therapist*" OR "radiology registrar*" OR "radiology trainee*" OR "radiologist trainee" OR "oncology registrar*" OR "oncology trainee*") |
| Intervention / Phenomenon of Interest (I) | Obesity / Larger Body Size | Controlled Vocabulary: MH Obesity+  Keywords: XB (obes*, overweight, BMI, “higher weight” "larger body habitus") |
| Comparison (C) | Not included | Not included |
| Outcome (O) | Weight bias, stigma, discrimination | Controlled Vocabulary: MH: (Attitude to Obesity), MH (Implicit Bias), MH (Weight Bias)  Keywords: XB (Bias* OR discriminat* OR stigma* OR stereotyp* OR victim* OR blam* OR sham* OR teasing OR unfair OR bully* OR harass* OR assumption* OR attribution* OR prejudice* OR perception* OR barrier* OR attitude* OR experience* OR belief* OR “anti-fat attitudes” or “fat shaming”) |
| Study Design / Research Type | Assessment / Measurement of weight bias | Controlled Vocabulary: MH (Measurement Issues and Assessments+)  Keywords: XB (attribution* OR prejudice* OR perception* OR barrier* OR attitude* OR experience* OR belief* OR “anti-fat attitudes” or “fat shaming” OR  ("fat phobia scale") OR ("anti fat attitudes scale") OR ("anti fat attitudes questionnaire*") OR ("belief about obese persons scale") OR ("attitude to obese persons scale") OR ("implicit association test" or iat)) |
